# Comparison between the Volatile Compounds and Physicochemical and Sensory Characteristics of Reverse-Seared and Conventionally Seared Beef Steaks

**DOI:** 10.3390/foods11142135

**Published:** 2022-07-19

**Authors:** In Young Lee, Boram Kim, Nami Joo

**Affiliations:** Department of Food and Nutrition, Sookmyung Women’s University, Seoul 04310, Korea; leakai@sookmyung.ac.kr (I.Y.L.); fanta-fun@sookmyung.ac.kr (B.K.)

**Keywords:** Maillard reaction, reverse searing, searing, steak, volatile compounds

## Abstract

This study was conducted to determine the effect of heat treatment (searing and reverse searing) on the volatile components and physicochemical and sensory characteristics of beef tenderloin. In reverse-seared steaks (RSSs), the loss rate was lower than in seared steak (SS), and several free amino acids (glutamic acid, glycine, histidine, and anserine) were increased, while a decrease in fatty acids (FAs) and volatile compounds (VCs) was observed. The VCs were identified as 24 compounds: 11 aldehydes, 5 alcohols, 1 ketone, 1 furan, and 6 aliphatic hydrocarbons. Hexanal was the predominant aldehyde, followed by pentanal and heptanal in both groups. Among the VCs with significant differences, only hexadecanal and 2,3-octanedione were increased in RSSs. During texture profile analysis (TPA), it was found that the characteristics of hardness, chewiness, and gumminess were lower in RSSs, which resulted in a higher score in the sensory evaluation. In conclusion, it was confirmed that the reverse searing method reduced the formation of meat flavor more than the searing method due to the limited Maillard reaction it caused. However, the mild heat treatment in the reverse searing process caused a remarkable increase in the appearance, texture, and amino acid content, which positively influenced the flavor.

## 1. Introduction

Heat treatment can significantly change meat’s composition, as well as its flavor components and physicochemical properties. When meat composition is combined with a specific cooking methodology, it majorly affects the final quality of meat products [1]. Furthermore, heat treatment can result in decreased nutritional value because of the oxidative modifications of proteins, which finally leads to decreased protein digestibility [2] and changes in fatty acid (FA) composition. Steak is generally sliced across the muscle fibers and grilled. Beef steaks can be cooked at different temperatures and lengths of time using dry heat, and the resulting cooked steaks can range from very rare to overdone. Searing means “burning” or “strongly scorching” and refers to a technique of cooking the surface of food at high temperatures to form a brown crust. The temperature of the meat’s surface should exceed 150 °C to attain the desired brown crust. Macleod [3] found that the primary pathway for non-volatile meat to generate flavor via heating is the oxidation and decomposition of fat and the pyrolysis and interaction of proteins, peptides, amino acids, sugars, and nucleotide target substances, and the secondary pathway is the Maillard reaction. It was reported that the searing process using the Maillard reaction secures the surface of the meat and reduces the loss of moisture, fat, and cooking due to the brown skin formed on the surface of the steak [4]. Reverse searing is the opposite method to strong searing and involves cooking meat evenly in an oven. This means that meat is slowly cooked in an oven at a relatively low temperature compared with searing and then seared over strong heat to finish. Recent reports suggest that reverse seared steak (RSS) has a more tender and juicy texture than conventionally seared steak (SS) and is, therefore, preferred by some meat lovers and chefs. The complicated relationship between texture and altered tenderness during cooking is regulated by the interactions between heat and time in various components, such as myofibrillar proteins, connective tissues, and the lipid matrix. It was learned in previous studies that protein denaturation and the loss of water-holding capacity within the meat are a result of dry cooking [5]. Therefore, pan-fried meat is initially softer but increases in toughness during prolonged cooking [6]. However, the differences between RSS cooked at low temperatures for an extended duration and conventionally SS have yet to be reported. Thus, this study was conducted to determine the effect of heat treatment (searing and reverse searing) on the volatile compounds (VCs) and physicochemical and sensory characteristics of beef tenderloin.

## 2. Materials and Methods

### 2.1. Sample Preparation

Frozen beef tenderloins (Choice, Excel Fresh Meats, Wichita, KS, USA) were purchased from a domestic food market (Daon Meat, Seoul, Korea), and sliced to 2 cm thicknesses, weighing 100 ± 5 g each. Before the heat treatment, the samples were stored in a refrigerator at 4 °C for 24 h. The samples were randomly divided into two groups: One group was used to prepare SS, and the other group was used to prepare RSS. Thirty beef tenderloins were randomly assigned to each group. The whole experiment was performed in triplicate using different carcasses each time.

### 2.2. Cooking Treatment

Cooking treatment methods were standardized through numerous preliminary experiments prior to analysis. To cook SSs, the beef samples were cooked on a pre-heated pan (200 ± 2 °C) for 2 min on both sides and then placed in a 180 °C pre-heated oven (ML32AW1, LG Electronics, Seoul, Korea) until the meat reached a 54.4 °C internal temperature (without flipping). On the other hand, RSSs were cooked in a 100 °C pre-heated oven (ML32AW1, LG Electronics) until the internal temperature reached 45 °C (without flipping), and then, the whole surface was evenly cooked on a pre-heated pan (200 ± 2 °C) for 45 s on both sides until the internal temperature reached 54.4 °C. Many experts recommend that the core of food should reach at least 54.4 °C, as all the known food pathogens stop growing and die when the food temperature exceeds about 52.3 °C [7]. Thus, the experiment was designed to ensure that the core temperature of the steaks reached 54.4 °C. Internal and surface temperatures of the samples were determined using a digital thermometer (MTC-701, Innpitron, Seoul, Korea) and an infrared thermometer (830-T2, Testo, Lenzkirch, Germany).

### 2.3. Determination of Water Content and Cooking Loss

The cooked beef steak was ground to measure the whole water content. The water content was measured with a moisture analyzer (MB45, Ohaus, Switzerland).

Beef (100 ± 5 g) was cooked, and after cooling at 20 °C for 1 h, the cooking loss was measured and calculated using the following formula: Cooking loss (%) = (Weight of the beef before cooking − Weight of the beef after cooking)/Weight of the beef before cooking × 100.

### 2.4. Determination of Color

The outside color of the sample was measured to determine the surface color (L*, a*, b* values). Additionally, the inside color was assayed at internal cross sections after cutting the steak. The color of each beef sample was measured with a colorimeter (CR-300, Minolta CO, Tokyo, Japan) and a standard (L* = 89.29, a* = −0.58, b* = 4.04). ΔE was calculated using the formula ΔE*_ab_ =$\sqrt{{\left(L^{*}{}_{2}-L^{*}{}_{1}\;\right)}^{2}+{\left(a^{*}{}_{2}-a^{*}{}_{1}\right)}^{2}+{\left(b^{*}{}_{2}-b^{*}{}_{1}\right)}^{2}}$.

### 2.5. Determination of Texture Profile Analysis (TPA)

The instrumental texture profile (hardness, adhesiveness, springiness, chewiness, gumminess, and cohesiveness parameters) was determined using a texture analyzer (TA-XT Express 2.1, London, Godalming, UK) with a cylinder probe that was 2.5 mm in diameter. A double compression cycle test was performed using a 25 kg load cell. The texture analyzer settings were as follows: pre-test speed, 2 mm/s; test speed, 1 mm/s; distance, 5 mm; trigger, 10 g; time, 5 s. Three steaks were assigned to each group, and four samples were cut from a steak. Nine of them were selected and analyzed. The samples were cut into 2 × 2 × 2 cm pieces.

### 2.6. Determination of Free Amino Acids

The analysis of free amino acids was performed as described by Cordoba et al. [8].

Each sample (1 g) was homogenized with 10 mL of ultrapure water and heated in a boiling water bath to solidify the sample. After that, the water layer was collected via filtration, and the fat was removed via extraction with ether. The aqueous layer was concentrated to dryness under reduced pressure. The obtained residue was dissolved in a 0.2 M citrate buffer (pH 2.2), filtered, and used as a sample. The free amino acid contents were determined using an amino acid analyzer (L8800, Hitachi, Tokyo, Japan). The amino acid analyzer conditions were as follows: column size, ion exchange resin #2622PF, column flow, 4.6 mm I.D. × 60 mm, ID 3 mm; 0.35 mL/min, ninhydrin 0.3 mL/min; temperature, main column 38~70 °C, reaction column 135 °C; injection volume, 20 μL; excitation, 440 nm; emission, 570 nm. The amino acid mixture Standard Solution Type AN-II, Type B (AA-S-18, Sigma, St. Louis, MO, USA) was used for the identification and quantification of free amino acids. The free amino acids are expressed as milligrams per 100 grams of the meat product.

### 2.7. Determination of Fatty Acids

The FA methyl esters (FAMEs) were determined from the extracted lipids as described in Kim et al. [9]. Each extracted sample (20 mg) was saponified with 0.5 M methanolic sodium hydroxide at 85 °C for 10 min and then methylated with 14% boron trifluoride in methanol. The samples were added to 3 mL of isooctane and 1 mL of saturated sodium chloride solution. The upper layer containing FAMEs was passed through an anhydrous sodium sulfate column. The FAMEs were analyzed on a gas chromatograph (Agilent 7890-A, Palo Alto, CA, USA). The FA analyzer conditions were as follows: column, 100 m × 0.25 mm i.d. × 0.2 μm film thickness (Supelco SP-2560, Bellefonte, PA, USA); carrier gas, He; column flow, 1 mL/min; injector temperature, 225 °C; detector temperature, 285 °C. The FAMEs were identified through a comparison of the retention times with those of reference standards (Supelco 37 component FAME Mix). The FAs are expressed as milligrams per 100 grams of the meat product.

### 2.8. Determination of Volatile Compounds

The VCs of the samples were determined using solid-phase microextraction (Headspace (HS)-SPME). A Supelco (Bellefonte, PA, USA) device containing fused silica fiber (10 mm length) coated with a 50/30 µm thick divinylbenzene/carboxen/polydimethylsiloxane (DVB/CAR/PDMS) was used. For HS-SPME extraction, each ground sample (5 g) was placed in a 20 mL vial. The fiber was inserted through the septum into the vial and exposed to sample headspace for 50 min at 80 °C. VCs were analyzed with a gas chromatograph (Agilent 7890/5975 MSD, Palo Alto, CA, USA). The VC analyzer conditions were as follows: column, 30 m × 0.25 mm i.d. × 0.25 μm film thickness (Agilent J&W, Palo Alto, CA, USA); carrier gas, He; column flow, 1 mL/min; aux temperature, 250 °C; MS source temperature, 230 °C; MS quad temperature, 150 °C; mass range, 35–500 *m*/*z*. The VCs were identified by comparing their mass spectra with those in NIST.

### 2.9. Sensory Analysis

The prepared samples were cut into slices (3 × 3 × 2 cm) with randomly selected 4-digit numbers. Twelve panelists who had more than one year of meat sensory evaluation experience and had previously been trained three times with standard beef steaks participated in the sensory evaluation. For the sensory analysis, preferences of color, appearance, juiciness, texture, umami taste, flavor, and overall quality were measured using a 7-point scale (from 1—extremely dislike to 7—extremely like). The sensory analysis was conducted over three independent sessions at different times. The sensory evaluation was performed following approval by the Institutional Review Board (IRB) of Sookmyung Women’s University (SMWU-2106-HR-052).

### 2.10. Statistical Analysis

The results are expressed as the mean ± S.D. of three separated determinations. Data sets were evaluated via a *t*-test using SPSS version 25 (SPSS Institute, Chicago, IL, USA) at a significance level of 0.05. The results regarding VCs were submitted to principal component analysis (PCA) using JMP^®^ Software (JMP^®^, Version 16.0, SAS Institute, Cary, NC, USA) to compare the composition of the SSs and RSSs.

## 3. Results

### 3.1. Water Content and Cooking Loss

The juiciness of steak is an important factor in the evaluation of meat quality; it was reported in a previous study that the juiciness was decreased in the sensory analysis following increased water loss when cooking steak [10].

In this study, water contents and cooking losses were significantly (*p* < 0.01) affected by the searing treatment (Table 1). Cooking losses were significantly higher when using the searing method due to the loss of the water-holding capacity of the proteins as they were denatured by the higher heat. The higher water content and lower cooking loss rate in RSS could increase the juiciness.

### 3.2. Color

The brown color of steak is caused by non-enzymatic browning reactions, which are the Maillard reaction and caramelization [11]. Therefore, the browning related to this factor was observed using the visual color of the steak.

The color parameters of SSs and RSSs are shown in Table 1. As regards surface color, RSSs had lower L*, a*, and b* values than SSs. The surface color was changed following searing; RSSs were darker with brown crusts. A similar decrease in L* and b* values associated with the darkening of the meat’s surface during prolonged cooking has been previously described [12].

### 3.3. TPA Values

The texture is a predominant element that determines the acceptability and quality of meat products. The TPA values of the samples prepared are shown in Table 1. In our study, hardness, adhesiveness, chewiness, and gumminess values were significantly different between the treatments. Adhesiveness in RSSs was higher than in SSs, and the hardness, chewiness, and gumminess values were lower in RSSs. However, there was no difference (*p* > 0.05) in springiness and cohesiveness.

### 3.4. Free Amino Acids

Not only do free amino acids contribute to taste, but they are also the precursors of volatile compounds formed via hydrolysis and the chemical transformation of Strecker degradation [13]. While some free amino acids directly improve taste, there are other amino acids that contribute to key aromatic compounds as precursors in beef steak. For example, glutamic acid and aspartic acid produce the umami taste, while leucine, phenylalanine, isoleucine, valine, methionine, and arginine form bitter flavors, and sour flavors are related to aspartic acid and histidine [14,15].

In this study, 19 free amino acids were identified, and their contents are shown in Table 2. Carnosine was the most abundant free amino acid, followed by taurine, anserine, alanine, and glutamic acid. Carnosine is a dipeptide of β-alanine and L-histidine and exhibits antioxidant, antiaging, and hypoglycemic effects; it is known to improve exercise performance and relieve muscle fatigue by neutralizing lactic acid produced during anaerobic glycolysis [16]. Taurine is a critical free amino acid that supports infant development and prevents medical conditions such as cardiovascular disease, hypertension, and diabetes [17]. Additionally, increased glutamic acid content contributes to the “umami” flavor of the meat. In addition, it increases the neuron fibers, detoxifies copper, enhances leukocyte activity, enhances immunity, and promotes insulin secretion [18]. The concentration of sulfur-containing amino acids, such as methionine, which are crucial for producing “meaty” odors after the Maillard reaction, only accounted for 3.3 mg and 3.57 mg of the total amino acid content in SSs and RSSs, respectively.

### 3.5. Fatty Acids

Thermal treatments can affect FAs among the lipid compounds of meats [1], and in particular, polyunsaturated FAs (PUFAs) are more highly susceptible to oxidation during heating processes than their saturated analogues, which may change the nutritional value of cooked products [19]. The influence of heat treatments on the composition of FAs is shown in Table 3. In the decreasing order of percentage, the major FAs in beef were found to be cis elaidic (18:1 (n-9)c), palmitic (16:0), stearic (18:0), and linoleic (18:2 (n-6)c) acids. Depending on the cooking conditions, the fat extracted from RSSs contained lower levels of saturated FAs (SFA) than SSs (12.97%). Reverse searing decreased the palmitic acid (16:0) and stearic acid (18:0) content in the fat extracted from beef by 9.64% and 14.07%, respectively. As expected, unsaturated FA (UFA) contents were lower in RSSs than in SSs. However, the levels of UFAs such as arachidonic acid (20:4 (n-6)), dihomo-γ-linolenic acid (20:3 (n-6)), and γ-linolenic acid (18:3 (n-6)) were increased in RSSs (7.26%, 5.30%, and 49.53%). Several mechanisms that occur during cooking, such as lipid oxidation and water loss, can lead to changes in PUFAs [19]. All of the levels of fatty acids (SFA, MUFA, PUFA, and TFA), except for 18:3 (n-6), 20:4 (n-6), 20:3 (n-6), 20:5 (EPA), and 22:5 (DPA), were increased in SSs rather than in RSSs, possibly due to the fact that they were cooked at a high temperature. However, within the 27 FAs analyzed, no significant differences were observed, except for in arachidic (20:0), behenic (22:0), and dihomo-γ-linolenic (20:3 (n-6)) acids.

### 3.6. Volatile Compounds

Altering FAs’ composition in meat directly affects the VCs formed during cooking [20]. Particularly, the autoxidation of FAs correlates with the formation of aldehydes, furans, and organic acids such as hexanoic acid [21]. Among the VCs, furan and pyrazine are particularly important contributors to enhancing the flavor of beef [22].

A total of 24 VCs were identified and classified according to their chemical nature (Table 4: 11 aldehydes, 5 alcohols, 1 ketone, 1 furan, and 6 aliphatic hydrocarbons. Hexanal was the predominant aldehyde, followed by pentanal and heptanal. Other aldehydes, such as nonanal, octanal, and benzaldehyde, were found to be less prevalent than other aldehydes. Aldehydes are the dominant chemical group detected in cooked meat samples of ruminants. In addition, aldehydes are known to be mostly derived from PUFA oxidation during heat treatment and amino acids’ degradation during the Maillard reaction through Strecker degradation [23]. Octane-2-3-dione was the main ketone in both samples. Biller [24] reported that octane-2-3-dione is formed in the Maillard reaction of lysine and ribose during prolonged heating, which supports our findings. Furan derivatives are typical compounds formed in the Maillard reaction and fat degradation [22]. In both samples, only a single furan was identified.

**Figure 1:**
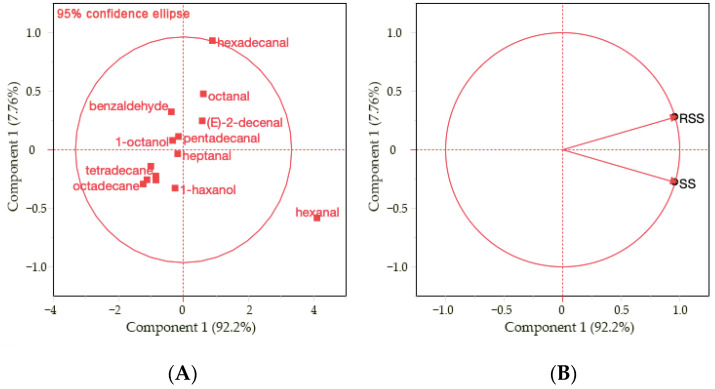
Score plot (**A**) and a loading plot (**B**) of principal component analysis (PCA) of 14 significant VCs found in seared and reverse-seared steaks.

Among the analyzed VCs, 14 components showed significant differences between samples. Therefore, principal component analysis (PCA) was performed to determine the relationship between the VCs in steaks (Figure 1). The two principal components accounted for 92.2% and 7.76%, respectively (99.96% in total). Aldehydes, such as hexanal, octanal, (E)-2-decenal, benzaldehyde, pentadecanal, and hexadecanal, were found to be positively related to PC1, while aliphatic hydrocarbons, such as tetradecane, hexadecane, and octadecane, were clustered together and negatively related to PC1. In the loading plot (Figure 1A), a clear separation between samples can be observed along PC2. Higher levels of VCs, such as aldehydes, alcohols, furans, and aliphatic hydrocarbons, were observed in SSs (hex-anal, octanal, (E)-2-decenal, pentadecanal, 1-hexanol, heptanal, 1-octanol, 2-pentylfuran, tetradecane, and hexadecane) than in RSSs. In particular, higher amounts of hexanal were observed in SSs, which is a degradation product of n-6 polyunsaturated FAs [25]. Hexanal can also be obtained via the non-enzymatic browning reaction due to the absence of fat in the matrix [24]. The level of octane-2-3-dione increased in RSSs, with higher lysine contents following prolonged exposure to heat. Overall, there was a significant difference in volatile compounds between both samples, and the reverse searing treatment decreased the formation of VCs associated with oxidation.

### 3.7. Sensory Evaluation

The sensory properties achieved with different heat treatments were evaluated by the trained panel. As a result, the RSS scored higher in all parameters (Table 5). The mild heat treatment in the reverse searing process induced a remarkable improvement in appearance and texture, which resulted in an increase in overall quality. Under extended heat exposure, the umami taste was increased by using the low heating temperature (80–100 °C); however, it was obviously decreased when the temperature rose above 120 °C, and it was replaced by a bitter flavor [26]. In this study, the RSS obtained higher scores in terms of the umami taste in the sensory evaluation. Quantitative studies of free amino acids showed that the glutamic acid content increases rapidly at low temperatures and is an important contributor to the umami taste [26]. In terms of flavor, the SS scored higher, presumably due to the higher volatile content, but there was no significant difference.

## 4. Conclusions

The current study shows that higher temperatures increase the formation of meat flavor in the Maillard reaction model for seared beef steak, whereas the umami taste is attributed to relatively lower temperatures and longer heating times. Quantitative studies of free amino acids showed that glutamic acid increases rapidly at low temperatures, and this component is considered to be an important contributor to the umami taste. The analysis of VCs showed that hexanal was the predominant aldehyde in the Maillard reaction and was significantly increased using the searing method. The findings of this study revealed that the loss rate was decreased during reverse searing and that juiciness somewhat increased, compared with conventional SS. Accordingly, the hardness, chewiness, and gumminess ratings were lower in RSSs, which resulted in a higher score in the sensory evaluation. In conclusion, it was confirmed that the reverse searing method reduced the formation of meat flavor to a greater extent than the searing method due to the limited Maillard reaction. However, the mild heat treatment in the reverse searing process caused a remarkable increase in the appearance, texture, and amino acid content, which positively influenced the meat quality.

## Figures and Tables

**Table 1:** Water content (%), cooking loss (%), CIELab (L*a*b*) color, and TPA values of seared and reverse-seared steaks (means ± SD).

		Seared Steak	Reverse-Seared Steak	t
Water content		57.21 ± 0.08 ^a^	58.63 ± 0.08 ^b^	−22.76 ***
Cooking loss		23.56 ± 1.06 ^a^	18.43 ± 0.55 ^b^	7.46 **
Surface color	L*	20.55 ± 1.68 ^a^	13.86 ± 0.93 ^b^	6.04 **
	a*	9.86 ± 1.90	8.60 ± 0.57	1.09
	b*	7.84 ± 0.43 ^a^	5.21 ± 0.43 ^b^	7.49 **
	ΔE	24.12 ± 2.34 ^a^	17.32 ± 1.05 ^b^	4.59 *
Inside color	L*	31.97 ± 0.73	33.78 ± 4.58	−0.68
	a*	26.35 ± 2.49 ^a^	19.92 ± 2.10 ^b^	3.42 *
	b*	10.13 ± 0.52 ^a^	8.73 ± 0.24 ^b^	4.27 *
	ΔE	42.67 ± 1.88	40.27 ± 3.69	1.00
Hardness (N/m^2^)		574.60 ± 11.92 ^a^	481.40 ± 17.63 ^b^	7.59 **
Adhesiveness (g × s)		−2.83 ± 0.64 ^a^	−0.43 ± 0.40 ^b^	−5.52 **
Springiness (mm)		0.80 ± 0.04	0.74 ± 0.03	2.08
Chewiness (N × mm)		272.10 ± 58.02 ^a^	161.97 ± 29.68 ^b^	2.93 *
Gumminess (N)		386.48 ± 34.14 ^a^	273.77 ± 56.86 ^b^	2.94 *
Cohesiveness (%)		0.51 ± 0.02	0.53 ± 0.06	−0.74

Values are presented as mean ± SD (*n* = 9); *, **, *** indicate significant differences as *p* < 0.05, *p* < 0.01, and *p* < 0.001, respectively; different letters indicate significant differences (*p* < 0.05).

**Table 2:** Free amino acids (mg/100 g) in seared and reverse-seared steaks.

Free Amino Acids	Seared Steak	Reverse-Seared Steak	t
Taurine	90.4 ± 45.66	111.67 ± 13.05	−0.78
Aspartic acid	0.13 ± 0.23	0.53 ± 0.11	−2.50
Threonine	4.67 ± 0.67	5.97 ± 0.45	−2.76
Serine	5.90 ± 1.30	7.07 ± 0.55	−1.42
Glutamic acid	13.70 ± 0.98 ^a^	17.67 ± 1.55 ^b^	−3.73 *
Glycine	8.00 ± 1.14 ^a^	10.47 ± 0.35 ^b^	−3.59 *
Alanine	28.53 ± 3.64	34.70 ± 2.16	−2.50
Valine	5.37 ± 1.46	6.87 ± 0.50	−1.69
Methionine	3.37 ± 1.93	3.57 ± 0.05	−0.18
Isoleucine	3.73 ± 1.62	4.43 ± 0.15	−0.75
Leucine	7.33 ± 3.27	8.50 ± 0.35	−0.62
Tyrosine	4.03 ± 1.70	4.77 ± 0.25	−0.74
Phenylalanine	4.77 ± 2.02	5.87 ± 0.10	−0.94
Ornithine	1.7 ± 0.52	1.43 ± 0.05	0.88
Lysine	4.93 ± 0.76	6.30 ± 0.45	−2.67
Histidine	3.27 ± 0.06 ^a^	3.93 ± 0.20 ^b^	−5.35 **
Anserine	77.63 ± 15.70 ^a^	105.80 ± 5.95 ^b^	−2.81 *
Carnosine	265.97 ± 49.00	277.53 ± 13.83	−0.37
Arginine	5.27 ± 0.47	6.03 ± 0.55	−1.80

Values are presented as mean ± SD (*n* = 3); *, ** indicate significant differences as *p* < 0.05 and *p* < 0.01, respectively; different letters indicate significant differences (*p* < 0.05).

**Table 3:** Fatty acids (mg/100 g) of seared and reverse-seared steaks.

Fatty Acid	Seared Steak	Reverse-Seared Steak	t
10:0	6.19 ± 0.55	5.54 ± 0.36	1.69
12:0	7.99 ± 0.67	7.05 ± 0.35	2.15
14:0	334.18 ± 33.88	300.01 ± 18.52	1.53
15:0	66.76 ± 6.94	58.57 ± 3.86	1.79
16:0	2800.68 ± 281.69	2530.79 ± 160.98	1.44
17:0	171.61 ± 19.02	156.80 ± 10.39	1.18
18:0	2238.6 ± 249.22	1923.54 ± 131.62	1.94
20:0	17.05 ± 1.50 ^a^	14.05 ± 0.92 ^b^	2.95 *
22:0	10.07 ± 0.72 ^a^	8.05 ± 0.66 ^b^	3.59 *
23:0	3.04 ± 0.31	2.54 ± 0.13	2.59
24:0	7.22 ± 1.89	5.38 ± 0.63	1.60
14:1	31.27 ± 2.94	29.56 ± 1.78	0.87
16:1	214.24 ± 20.21	204.39 ± 12.11	0.72
18:1t (n-9)	717.53 ± 78.61	614.28 ± 38.54	2.04
18:1 (n-9) _c_	3305.82 ± 344.54	2977.3 ± 180.80	1.46
18:1 (n-7) _c_	143.63 ± 12.77	130.43 ± 7.77	1.53
18:2t (n-6)	62.82 ± 5.03	55.01 ± 3.23	2.26
18:2 (n-6) _c_	1006.54 ± 68.34	936.05 ± 53.23	1.41
18:3(n-6)	1.52 ± 1.31	2.27 ± 0.09	−0.21
18:3t	17.05 ± 3.12	15.59 ± 0.33	0.80
20:1	20.78 ± 1.74	17.67 ± 1.54	2.32
18:3 (n-3)	80.67 ± 7.29	71.52 ± 3.91	1.92
20:2 (n-6)	4.60 ± 0.93	4.22 ± 0.16	0.70
20:3 (n-6)	27.21 ± 0.76 ^a^	28.65 ± 1.37 ^b^	−3.01 *
20:4 (n-6)	85.31 ± 3.58	91.51 ± 3.91	−2.03
20:5 (EPA)	5.55 ± 0.19	5.88 ± 0.39	−1.32
22:5 (DPA)	15.94 ± 0.75	16.39 ± 0.73	−0.74
SFA	5663.39 ± 591.03	5012.31 ± 328.10	1.67
MUFA	3715.74 ± 382.01	3359.34 ± 203.76	1.43
PUFA	1227.34 ± 71.54	1156.48 ± 63.62	1.28
TFA	797.39 ± 86.72	684.88 ± 42.01	2.02

Values are presented as mean ± SD (*n* = 3); * indicates significant differences as *p* < 0.05; different letters indicate significant differences (*p* < 0.05).

**Table 4:** Volatile compounds (%) of seared and reverse-seared steaks.

Volatile Compounds	Seared Steak	Reverse-Seared Steak	t
*aldehydes*			
hexanal	16.64 ± 0.45 ^a^	5.13 ± 0.01 ^b^	44.30 ***
octanal	3.25 ± 0.19 ^a^	2.73 ± 0.97 ^b^	4.07 *
nonanal	6.44 ± 0.11	6.41 ± 0.20	0.26
(E)-2-nonenal	1.45 ± 0.06	1.55 ± 0.10	−1.37
decanal	0.32 ± 0.02	0.39 ± 0.13	−0.87
(E)-2-decenal	3.83 ± 0.32 ^a^	2.47 ± 0.04 ^b^	45.62 ***
benzaldehyde	0.81 ± 0.12 ^a^	1.61 ± 0.05 ^b^	−10.76 ***
tridecanal	0.36 ± 0.20	0.38 ± 0.05	−0.69
tetradecanal	0.96 ± 0.15	0.89 ± 0.10	1.31
pentadecanal	2.08 ± 0.15 ^a^	1.62 ± 0.46 ^b^	16.38 ***
hexadecanal	2.73 ± 0.23 ^a^	3.45 ± 0.95 ^b^	−12.75 ***
*ketones*			
octane-2-3-dione	0.32 ± 0.15 ^a^	0.53 ± 0.75 ^b^	−4.60 *
*alcohols*			
1-hexanol	3.06 ± 0.65 ^a^	1.08 ± 0.04 ^b^	46.03 ***
heptanal	2.44 ± 0.26 ^a^	1.45 ± 0.08 ^b^	19.56 ***
1-heptanol	0.87 ± 0.12	0.75 ± 0.03	1.69
1-octen-3-ol	1.12 ± 0.31	1.11 ± 0.04	0.24
1-octanol	1.65 ± 0.25 ^a^	1.41 ± 0.06 ^b^	6.30 **
*furans*			
2-pentylfuran	1.13 ± 0.21 ^a^	0.57 ± 0.03 ^b^	4.64 *
*aliphatic hydrocarbons*			
dodecane	0.19 ± 0.17	0.19 ± 0.02	0.25
tridecane	0.49 ± 0.31	0.37 ± 0.16	1.33
tetradecane	1.02 ± 0.01 ^a^	0.60 ± 0.05 ^b^	14.29 ***
hexadecane	0.31 ± 0.03 ^a^	0.30 ± 0.13 ^b^	0.13 *
heptadecane	0.12 ± 0.15	0.19 ± 0.05	−2.21
octadecane	0.08 ± 0.00 ^a^	0.15 ± 0.02 ^b^	−5.55 **

Values are presented as mean ± SD (*n* = 3); *, **, *** indicate significant differences as *p* < 0.05, *p* < 0.01, and *p* < 0.001, respectively; different letters indicate significant differences (*p* < 0.05).

**Table 5:** Scores of sensory evaluations of seared and reverse-seared steaks.

Sensory Parameter	Seared Steak	Reverse-Seared Steak	t
Color	3.30 ± 0.82 ^a^	5.40 ± 0.52 ^b^	−6.83 ***
Appearance	4.00 ± 0.82 ^a^	5.20 ± 0.63 ^b^	−3.67 **
Juiciness	3.30 ± 0.67 ^a^	4.40 ± 1.07 ^b^	−2.74 *
Texture	4.20 ± 0.92 ^a^	5.80 ± 0.63 ^b^	−4.54 ***
Umami	3.50 ± 0.97 ^a^	4.90 ± 0.88 ^b^	−3.38 **
Flavor	4.20 ± 0.79	4.10 ± 0.74	0.29
Overall quality	3.70 ± 0.67 ^a^	4.90 ± 0.74 ^b^	−3.80 ***

Values are presented as mean ± SD (*n* = 12); *, **, *** indicate significant differences as *p* < 0.05, *p* < 0.01, and *p* < 0.001; different letters indicate significant differences (*p* < 0.05).

## Data Availability

Data are contained within the article.
